# Phenytoin versus Leviteracetam for Seizure Prophylaxis after brain injury – a meta analysis

**DOI:** 10.1186/1471-2377-12-30

**Published:** 2012-05-29

**Authors:** Syed Nabeel Zafar, Abdul Ahad Khan, Asfar Ayaz Ghauri, Muhammad Shahzad Shamim

**Affiliations:** 1 Department of Surgery, Aga Khan University, Karachi, Pakistan; 2 Medical College, Aga Khan University, Karachi, Pakistan

**Keywords:** Levetiracetam, Phenytoin, Meta-analysis, Brain injury, Seizures, Prophylaxis, Anti-epileptic drugs

## Abstract

**Background:**

Current standard therapy for seizure prophylaxis in Neuro-surgical patients involves the use of Phenytoin (PHY). However, a new drug Levetiracetam (LEV) is emerging as an alternate treatment choice. We aimed to conduct a meta-analysis to compare these two drugs in patients with brain injury*.*

**Methods:**

An electronic search was performed in using Pubmed, Embase, and CENTRAL. We included studies that compared the use of LEV vs. PHY for seizure prophylaxis for brain injured patients (Traumatic brain injury, intracranial hemorrhage, intracranial neoplasms, and craniotomy). Data of all eligible studies was extracted on to a standardized abstraction sheet. Data about baseline population characteristics, type of intervention, study design and outcome was extracted. Our primary outcome was seizures.

**Results:**

The literature search identified 2489 unduplicated papers. Of these 2456 papers were excluded by reading the abstracts and titles. Another 25 papers were excluded after reading their complete text. We selected 8 papers which comprised of 2 RCTs and 6 observational studies. The pooled estimate’s Odds Ratio 1.12 (95% CI = 0.34, 3.64) demonstrated no superiority of either drug at preventing the occurrence of early seizures. In a subset analysis of studies in which follow up for seizures lasted either 3 or 7 days, the effect estimate remained insignificant with an odds ratio of 0.96 (95% CI = 0.34, 2.76). Similarly, 2 trials reporting seizure incidence at 6 months also had insignificant pooled results while comparing drug efficacy. The pooled odds ratio was 0.96 (95% CI = 0.24, 3.79).

**Conclusions:**

Levetiracetam and Phenytoin demonstrate equal efficacy in seizure prevention after brain injury. However, very few randomized controlled trials (RCTs) on the subject were found. Further evidence through a high quality RCT is highly recommended.

## Background

Seizures in neurosurgical patients are a common occurrence and may lead to several potential complications such as higher metabolic demand of neurons, increased intracranial pressure and secondary brain injury. Anti-epileptic drug (AED) prophylaxis is commonly instituted for the management of patients with brain injury as certain sub-groups have been shown to have beneficial effects of seizure prophylaxis [1]. Compared to placebo, the drug Phenytoin (PHY) has been reported to be significantly more effective in preventing post traumatic seizures during the first 7 days (risk ratio, 0.27; 95 percent confidence interval, 0.12 to 0.62) [2]. However, Phenytoin displays a wide array of side effects including induction of the hepatic cytochrome P450 system, cutaneous hypersensitivity reactions and inducing drug-drug interactions [3,4]. Levetiracetam (LEV) on the other hand, is a relatively new non-enzyme inducing AED and is reported to have far lesser potential side effects [5]. Additionally, in contrast to PHY, it does not require close monitoring by serial blood sampling due to a wider therapeutic index. However, it is far more expensive than PHY. In a recent study the cost of a 7-day course of PHY was $37.50 compared to $480.00 for a 7 day course of LEV [6].

There is debate on the effectiveness of LEV compared to PHY in seizure prophylaxis. Various trials have shown diversified results with regards to the relative effectiveness of the two drugs. Jones et al. [7] noted similar efficacy for both drugs with regards to prevention of seizures after traumatic injury. However, LEV was attributed to increased epileptic activity on EEG monitoring. On the other hand, a randomized controlled trial [8] revealed better long term outcomes for LEV after neurosurgical injury compared to Phenytoin, with no difference in seizure occurrence during EEG. We aimed to conduct a meta-analysis of studies comparing the efficacy of these two drugs in patients with brain injury.

## Methods

### Search strategy

We systematically searched MEDLINE, EMBASE, ClinicalTrials.gov and the Cochrane Central Register of Controlled Trials (CENTRAL) for all comparative studies and conference abstracts comparing the effect of Phenytoin (PHY) to Levetiracetam (LEV) on seizure prophylaxis among patients with brain injury. We defined brain injury as patients with traumatic brain injury (TBI), intracranial hemorrhage or those undergoing a craniotomy for any reason. We constructed search filters for a) PHY (Phenytoin, Dilantin), b) LEV (Levetiracetam, Keppra) and c) brain injury (TBI, intracranial hemorrhage, intracranial neoplasms, craniotomy) using a combination of MeSH terms and text words searches for synonyms and related diseases. We separated these three concepts by the boolean “AND” and limited the results (wherever possible) by limiting to comparative studies (details of search terms are provided in Additional file 1). No limits were applied on language or date of publication. The search was performed first on February 2^nd^, 2011 and updated as of October 20^th^, 2011. Additionally we manually searched references of key articles. This meta-analysis is reported in accordance with the MOOSE guidelines [9]. Endnote X4 was used to maintain and manage our library.

### Selection criteria

In duplicate and independently two investigators (AK and AG) screened all studies and selected articles that satisfied the inclusion criteria; a) comparative study (trials, cohorts, case-controls and observational studies), b) the study population consisted of patients with brain injury, c) the study compared LEV to PHY and d) the study reported outcomes of seizures and/or side effects. We excluded studies that used combination therapies instead of LEV and PHY monotherapy unless there were separate arms for monotherapy. We aimed to include only randomized controlled trials (RCTs) in our analysis however due to their paucity we included other intervention studies (before-after) and observational studies. Disagreements were resolved by consensus or group discussion with a third author.

### Data abstraction

We extracted data from all eligible studies on to a standardized data abstraction sheet. The extraction was checked by another author independent of the first. We extracted information on study characteristics, characteristics of the population under study, operational definitions and outcomes. Emails were sent to the corresponding or first author of the studies or abstracts for missing information. We waited for responses from authors for a period of 12 weeks till October 1^st^ 2011. A reminder email was also sent during this period.

### Outcomes

Outcome data was collected for seizures (proportion of patients with early and late seizures) and side effects (presence/absence of individual side effects and presence/absence of any side effect studied by the authors). We defined ‘early’ seizures as the number of patients that had a seizure within a given time interval as defined by the author. When there were a number of time intervals we took it to be from injury till discharge or 30 days. Since there is no consensus on the definition of ‘early seizures’ we also performed a subset analysis defining ‘early seizures’ as seizures occurring within 7 days. ‘Late seizures’ was defined as the number of patients that had seized at a 6 month follow-up.

### Quality assessment

We used the ‘Newcastle-Ottawa Scale’ to assess the quality of studies selected for our analysis. This scale grades each study on three criteria; selection (maximum of four stars), comparability (maximum of 2 stars) and outcome assessment (maximum of 3 stars). This is the scale recommended by the Cochrane Non-Randomized Studies Methods Working Group.

### Statistical analysis

Our primary outcomes were early and late seizures and side-effects. We performed a meta-analysis when data was available for more than one study. The summary effect estimate used was the odds ratios with its 95% confidence interval. A 0.5 continuity correction was applied to all four cells in case a zero value was present. We used a DerSimonian and Laird random effects model with inverse variance weights to derive our pooled effect estimate and Forrest plots were generated. Between studies heterogeneity was assessed using a Cochran’s Q statistic and the I^2^ statistic [10]. We considered a p value ≤0.1 or an I^2^ value of 50% or more as evidence of heterogeneity. If heterogeneity was found we planned for subset analyses by follow up period for ‘early seizures’ and by eliminating one study at a time and rechecking the heterogeneity.

We assessed publication bias by the Egger test and visual inspection of the funnel plot [11]. We considered a p value of <0.05 as evidence of significant publication bias. All analyses were performed on STATA version 11 (STATA/SE, College Station, TX).

## Results

### Literature search and study characteristics

**Figure 1:**
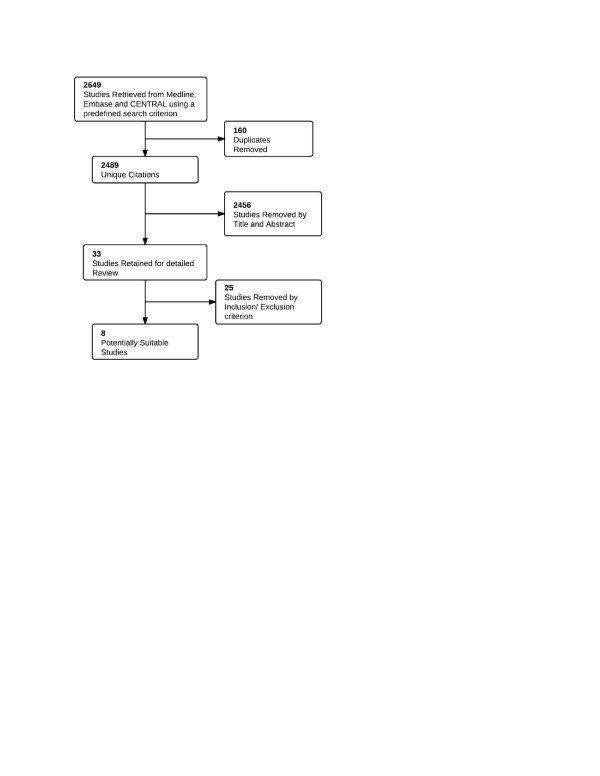
Flow chart for study selection.

Our search strategy initially identified 2,649 studies of which 2,489 were unique (Figure 1). After screening titles and abstracts we removed 2,456 studies and retrieved the full text of 33 studies. From these 8 studies (6 observational studies and 2 RCTs) were selected to be suitable for our meta-analysis and authors were contacted for further information if necessary [7,12-17]. Due to paucity of data we limited our analysis to only two outcomes; early and late seizures. 6 studies (4 observational and 2 RCTs) reported these outcome and were selected for our analysis [7,12-15].

The study characteristics are presented in Table 1. A total of 990 patients were included. All publications were recent (from 2008 to 2011) and were all conducted in the USA. The mean age of participants ranged from 33 years to 70 years. Most studies had a high percentage of males except the study by Murphey-Human et al. where both arms had around 70% females. All studies were of sufficient quality to be included in the analysis (Table 2). Only the study by Jones et al. had questionable comparability between the two arms as no method to adjust for confounders was used.

**Table 1:** Characteristics of studies included in the met-analyses

**Study**	**Country**	**Study Design**	**Population Type**	**Analyzed**		**Mean** **Age** (years)*		**% Males**		**Dosage/day**		**Seizures assessed at**
				**PHY**	**LEV**	**PHY**	**LEV**	**PHY**	**LEV**	**PHY**	**LEV**	
**Jones et al.**[7]	USA	Obs	Severe TBI	41	32	34.6	33.2	75	73	N/A	1000 mg	7 Days
**Milligan et al.**[12]	USA	Obs	Supra-tentorial surgery	210	105	60	56.3	47	39	200-800 mg	500-3000 mg	7 Days & 30 Days
**Lim et al.**[13]	USA	RCT	Glioma (Post Operative)	8	15	48.2	42	100	60	300-400 mg	1000-3000 mg	6 months
**Szaflarski**[8]	USA	RCT	TBI, SAH	18	34	42	45	72.2	76.5	5 mg/kg	2000-3000 mg	3 Days & 6 months
**Taylor et al.**[14]	USA	Obs	ICH	25	60	70.2	63.3	52	40	NA	500-2000 mg	Till discharge
**Murphy-Human et al.**[15]	USA	Obs	SAH	297	145	57	55	30	28	NA	1000 mg	3 Days & Till discharge

*Obs* Observational study, *RCT* Randomized Controlled Trial, *LEV* Levetiracetam, *PHY* Phenytoin, *TBI* Traumatic brain injury, *ICH* Intracranial hemorrhage, *SAH* Subarachnoid hemorrhage.

* Mean age has been estimated wherever not directly available.

**Table 2:** Quality assessment of studies included in the meta-anlayses

**Study**	**Study type**	**Selection**	**Comparability**	**Outcome/Exposure**
Jones et al [7]	Cohort	***		***
Milligan et al. [12]	Cohort	****	*	***
Lim et al. [13]	RCT	***	**	**
Szaflarski [8]	RCT	***	**	***
Taylor et al. [14]	Cohort	***	**	**
Murphy-Human et al. [15]	Cohort	***	**	***

### Outcomes

**Figure 2:**
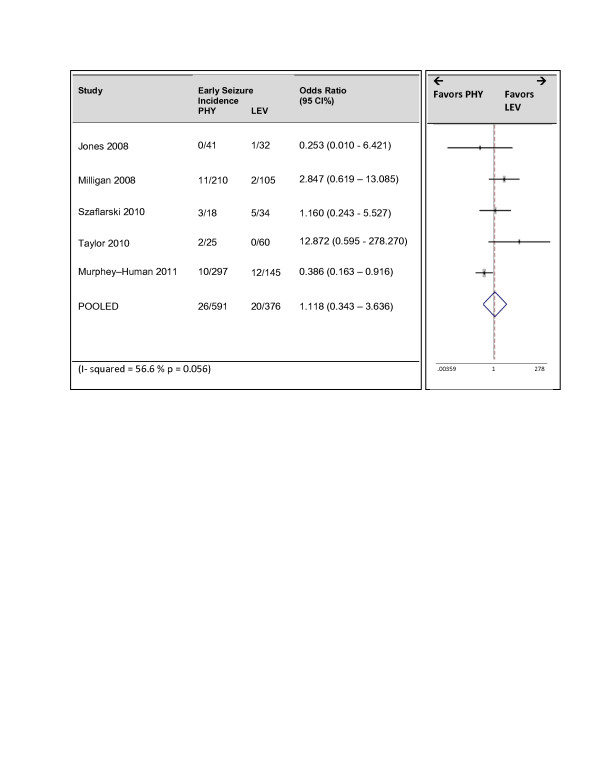
Forrest Plot of studies reporting early seizures.

**Figure 3:**
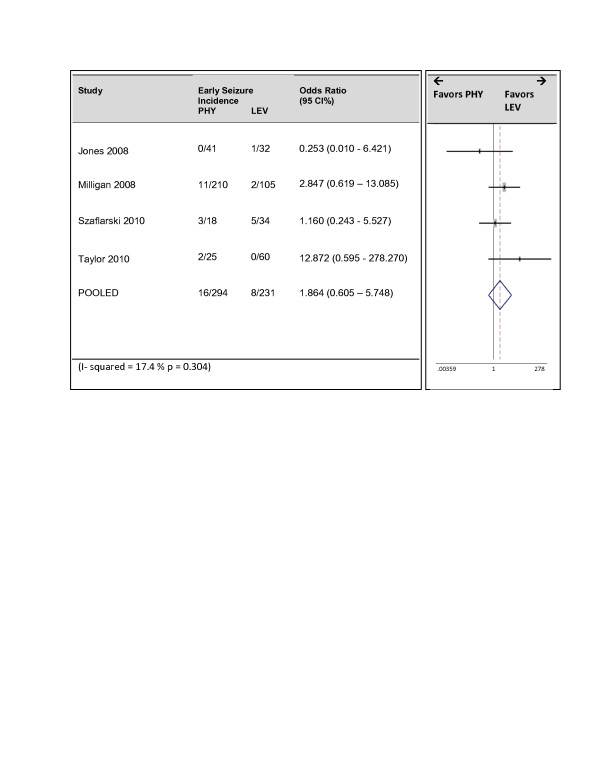
Subset analysis: Forrest Plot of studies reporting early seizures excluding the study by Murphey-Human et al.

Five of the six studies reported early seizures. Follow up times ranged from 3 to 30 days. The pooled estimate demonstrated no superiority of either drug at preventing the occurrence of early seizures (Figure 2). The pooled odds ratio was 1.12 (95% CI = 0.34, 3.64). However significant heterogeneity was found with a Cochran Q statistic p value of 0.056 and the I^2^ value was 57%. Upon sensitivity analysis, heterogeneity disappeared after removing the study by Murphey-Human et al. (I^2^ = 17%, p = 0.304). The pooled odds ratio of 1.9 (95% CI = 0.61, 5.75) favored less seizures in the LEV group however this estimate also remained insignificant (Figure 3). The study by Murphey- Human et al. was unique as it was the only study to demonstrate a difference in the two drugs, included only patients presenting with subarachnoid hemorrhage and also included a high proportion of females (70%). However it was also the largest study and rated highly on quality.

**Figure 4:**
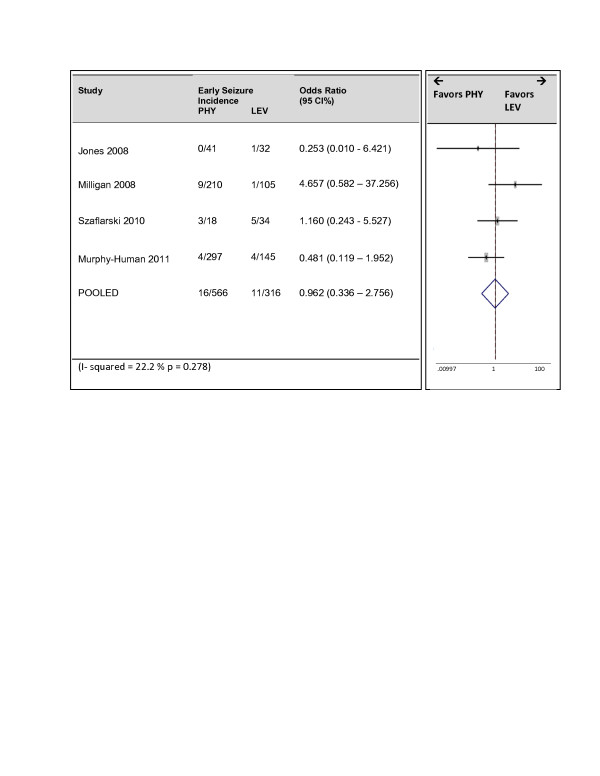
Subset analysis: Forrest Plot of studies reporting seizures within 7 days.

We performed a subset analysis of studies in which ‘early seizures’ was defined as seizures occurring within the first 7 days. Four studies were included in this analysis (Figure 4). Again heterogeneity was eliminated with the I^2^ being 22% (p = 0.278). The effect estimate remained insignificant with an odds ratio of 0.96 and 95% confidence bounds of 0.34 to 2.76.

**Figure 5:**
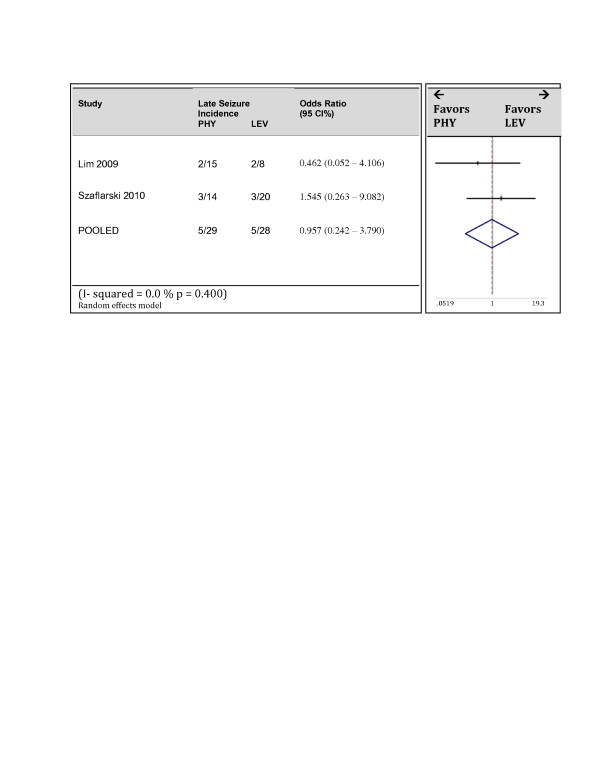
Forrest Plot of studies reporting late seizures.

Two studies reported seizure incidence at 6 months [8,13]. Both of these were randomized controlled trials. The pooled estimate again demonstrated no superiority of either drug (Figure 5). The pooled odds ratio was 0.96 with 95% confidence bounds of 0.24 and 3.79. The total number of patients however, was low with only 26 in the PHY arm and 49 in LEV arm. No heterogeneity was observed while pooling effects estimates of these two studies. The Cochran Q statistic p value equaled 0.400 and the I^2^ value was 0%.

We found no evidence of publication bias when we tested our primary outcome of ‘early seizures’. The Egger p value was 0.195 demonstrating no small study effects. The funnel plot is provided in Additional file 1.

## Discussion

We find no difference in the effectiveness of early or late seizure prophylaxis between Levetiracetam and Phenytoin in patients with brain injury. Seizure prophylaxis for neurosurgical problems has been in practice for a long time [18-23]. The practice is based on the understanding that various sub-groups of neurosurgical patients are at a relatively higher risk of seizures and onset of seizures has been shown to independently predict poor outcome [24-26]. The most common reason for risk of seizures in these patients is raised intracranial pressure and/or presence of an abnormal supratentorial focus; which may be the injured neural tissue itself, or an intra-cranial mass lesion such as a tumor. Supratentorial surgery also poses a similar risk for patients as post-operative cerebral edema from surgical manipulation and tissue trauma predisposes these patients to seizures [24,27,28]. It thus appeared reasonable to group such patients together for this meta-analysis, especially since the available literature on individual neurosurgical sub-groups does not provide sufficient patient numbers for conclusive scientific analysis.

Phenytoin has traditionally been the drug of choice for prophylaxis in these patients and even though its efficacy is widely accepted, the drug’s side effects remain a significant problem, especially on long term use [29-32]. Side effects from anti-epileptic medications are a serious problem in neurosurgical patients with data for brain tumor patients alone reporting severe side effects in up to 23.8% of patients [32-37]. These include drug-drug reactions, elevation of hepatic enzymes, skin related problems, thrombocytopenia, unexplained fever etc., and in one study resulted in discontinuation of Phenytoin therapy in 39.8% of patients [38]. Moreover, Phenytoin drug levels have to be periodically monitored to insure therapeutic levels in serum as small changes in drug dosage or metabolism may lead to disproportionate changes in serum concentrations [39].

Levetiracetam has been shown to have comparable clinical efficacy by a number of investigators, with the added benefit of much fewer side effects; and the fact that drug levels are not required to be serially monitored [38]. The few side effects associated with Levetiracetam include headache, nausea/vomiting, drowsiness, dizziness and behavioral changes. Milligan et al. in their study demonstrate that 64% of patients on Levetiracetam adhered to therapy after 12 month follow up as compared to 26% of patients on Phenytoin [12]. Levetiracetam was initially limited in its clinical application by a lack of available intravenous formulation, which hindered its usage in critically ill patients. Although this limitation has been overcome, the other potential disadvantage is the high cost of the drug, which makes its use difficult. Out of pocket payment systems in developing countries and limited discharge drug plans in developed countries make it a burden for the patient to bear this additional cost. In developed countries, limited drug plans upon discharge prove to be a hindrance for the patient to receive the medication. A recent study comparing the cost effectiveness of both drugs has estimated that for post-traumatic seizure prophylaxis, phenytoin costs $1.58 per quality adjusted life year (QALY) as compared to $20.72 per QALY for levetiracetam [6]. The authors concluded that levetiracetam can only be considered more cost-effective to phenytoin if it prevented 100% of seizures and costed < $400 for a 7 day course. However a limitation of this study is that it did not account for the costs related to monitoring of phenytoin blood levels or for the cost of side effects. The study assumed that ‘severe adverse events that could impact costs were rare for each drug’.

To date there have been several studies on the comparative efficacy and safety of these two drugs in different patient populations, although the numbers have been small and results variable [7,13,15]. Interestingly, all studies comparing the two drugs came from one region, North America. This is difficult to explain but may be either due to a larger number of practicing neurosurgeons and neurophysicians, wider interest in anti-epileptics, or better funding opportunities for drug related research. We nevertheless recommend more RCTs to be conducted in different parts of the world to provide a mix of population and eliminate bias.

Even though the benefits of seizure prophylaxis have been accepted for prevention of early seizures in TBI patients, there remains a question whether it should be used for other pathologies such as brain tumors and SAH; and for the prevention of late seizures. We did not attempt to evaluate the role of either of these drugs for individual pathologies, however, through this study; an attempt was made to separately analyze their efficacies in early and late seizures. The problem we encountered was the variations in the definition of early seizures. Since this was an analysis of published literature we were limited by the time interval in which each study assessed seizure activity. ‘Early seizures’ varied between 3 days, 7 days, 30 days or ‘till discharge’. Even though we found no heterogeneity in the results due to this variation, it remains a limitation of the study. To overcome this limitation, we conducted the analysis using two different types of definitions for ‘early seizures’; within the first month and within the first week. The results of both analyses were similar. We were able to study effects for a consistent definition of ‘late seizures’ (1 month to 6 months). Even though this may not be a typical definition of ‘late seizures’ it does provide us with a reliable measure to compare efficacy of the two drugs. Despite differences in the precise definition of early and late seizures, no statistically significant difference in risk of seizures could be found between the two drugs for either early, or late seizures.

We initially planned to do a meta-analysis of RCTs alone, however, a comprehensive search of literature failed to reveal adequate number of RCTs, or RCTs with large number of patients comparing these two drugs and we were therefore required to include observational studies in this analysis. This caused issues with comparability and adjustment of confounders. We would therefore recommend more RCTs on this topic, without which it remains elusive to reach meaningful conclusions in this regard. Since there were insufficient RCTs on the topic, we were required to include observational studies in the meta-analysis, with associated problems of comparability and confounders. This included the lack of standard dosage for the two drugs in individual studies. We were also not able to analyze all study details, despite our best efforts to contact authors of published or in print papers and abstracts. Similarly, we could not assess other outcomes such as drug side effects, number of seizures per patient, optimal dose and duration for prophylaxis.

## Conclusions

On the basis of our analysis of available literature, we conclude that there is no significant difference in seizure prophylaxis for either early or late seizures; for either Phenytoin or Levetiracetam. However, paucity of good quality evidence limits our conclusion. Better quality RCTs from centers in different parts of the world are recommended.

## Abbreviations

RCT: Randomized controlled trial; PHY: Phenytoin; LEV: Levetiracetam; CI: Confidence Interval; AED: Anti-epileptic drug; EEG: Electroencephalography; MeSH: Medical subject heading; MOOSE: Meta-analysis of observational studies in epidemiology.

## Competing interests

The authors declare that they have no competing interests.

## Authors’ contributions

SNZ and SS conceived of the study. SNZ and AAK finalized the search strategy. AAK and AAG primarily screened studies and abstracted data with supervision from SNZ and SS. SNZ, AAK and AAG analyzed the data. All authors took part in data interpretation, manuscript writing and critically reviewing the manuscript. All authors read and approved the final manuscript.

## Source of funding

This study did not receive any funding. None of the authors received funding in part or in full for contribution to this study or for any related work.

## Pre-publication history

The pre-publication history for this paper can be accessed here:

http://www.biomedcentral.com/1471-2377/12/30/prepub

## Supplementary Material

Additional file 1 **Annex 1**. Search Strategy, **Annex 2**. Funnel plot for publication bias. Click here for file
