# Supplementary material for: Phenytoin versus Leviteracetam for Seizure Prophylaxis after brain injury – a meta analysis
Source: BMC Neurol. 2012 May 29;12:30. doi: 10.1186/1471-2377-12-30 (PMC3406949; doi:10.1186/1471-2377-12-30)
Supplement: Additional file 1 — Annex 1. Search Strategy, Annex 2. Funnel plot for publication bias. [file 1471-2377-12-30-S1.doc]

**Annexes**

**Annex 1 – Search Strategy**

**Pubmed**

("Phenytoin"[Mesh] OR "Phenytoin" OR "Dilantin") AND ("etiracetam "[Substance Name] OR "Levetiracetam" OR "Keppra") AND ("Brain Injuries"[Mesh] OR (Brain AND injur*) OR "TBI*" OR (Head AND Injur*) OR "Cerebrovascular Trauma"[Mesh] OR "Cerebrovascular Trauma" OR "Epilepsy, Post-Traumatic"[Mesh] OR "Post Traumatic Epilepsy" OR "Brain Hemorrhage, Traumatic"[Mesh] OR "Traumatic Brain Hemorrhage" OR "Craniotomy"[Mesh] OR "Craniotomy" OR "Postoperative Period"[Mesh] OR "Post Operative Period" OR "Postoperative Care"[Mesh] OR "Postoperative care" OR "Postoperative Complications"[Mesh] OR "Postoperative Complications" OR "Brain Neoplasms"[Mesh] OR "Brain Neoplasms" OR "Glioma"[Mesh] OR "Glioma*" OR "Hemorrhage"[Mesh] OR "Hemorrhage*" OR "Intracranial Hemorrhages"[Mesh] OR "Intracranial Hemorrhage*" OR "Cerebral Hemorrhage"[Mesh] OR "Cerebral Hemorrhage*" OR "Subarachnoid Hemorrhage, Traumatic"[Mesh] OR "Traumatic Subarachnoid Hemorrhage*" OR “Subarachnoid Hemorrhage“[Mesh] “Subarachnoid Hemorrhage” "Cerebral Hemorrhage, Traumatic"[Mesh] OR "Traumatic Cerebral Hemorrhage*" OR "Cerebrovascular accident" "Stroke"[Mesh] OR "Stroke*" OR "Intracranial Aneurysm"[Mesh] OR "Intracranial Aneurysm*" OR "Epilepsy"[Mesh] OR "Seizures"[Mesh] OR "Seizure*" OR Epileps* OR “Brain Diseases” [Mesh] OR meningioma[Mesh] OR meningioma)

**Embase**

(Phenytoin OR Phenytoin/exp OR ‘Dilantin’) AND (‘etiracetam’ OR ‘etiracetam’/exp ‘Levetiracetam’ OR ‘Keppra’) AND (‘brain injury’ OR 'brain injury'/exp OR ‘head injury’ OR 'head injury'/exp OR ‘traumatic brain injury’ OR 'traumatic brain injury'/exp OR ‘cerebrovascular accident’ OR 'cerebrovascular accident'/exp OR ‘traumatic epilepsy’ OR 'traumatic epilepsy'/exp OR ‘subarachnoid hemorrhage’ OR 'subarachnoid hemorrhage'/exp OR ‘brain hemorrhage’ OR 'brain hemorrhage'/exp OR ‘craniotomy’ OR 'craniotomy'/exp OR ‘craniectomy’ OR 'craniectomy'/exp OR ‘post operative’ OR 'postoperative care'/exp OR ‘postoperative complication’ OR ‘postoperative complication’/exp OR ‘brain tumor’ OR 'brain tumor'/exp OR ‘meningioma’ OR ‘meningioma’/exp OR ‘glioma’ OR 'glioma'/exp OR ‘intracranial aneurysm’ OR 'intracranial aneurysm'/exp OR ‘epilepsy’ OR 'epilepsy'/exp OR ‘seizure’ OR 'seizure'/exp OR 'brain disease'/exp OR ‘brain disease’)

**Annex 2 – Funnel plot for publication bias**
